# *Geum sunhangii* (Rosaceae), a new species from Hubei Province, China

**DOI:** 10.3897/phytokeys.156.37277

**Published:** 2020-08-21

**Authors:** Zhen-Yu Lv, Dai-Gui Zhang, Xian-Han Huang, Heng-Chang Wang5, Jing-Yuan Yang6, Komiljon Tojibaev, Tao Deng, Zhi-Min Li

**Affiliations:** 1 School of Life Sciences, Yunnan Normal University, Kunming 650500, China; 2 Key Laboratory of Plant Resources Conservation and Utilization, Jishou University, Jishou, Hunan 416000, China; 3 Key Laboratory for Plant Diversity and Biogeography of East Asia, Kunming Institute of Botany, Chinese Academy of Sciences, Kunming 650201, Yunnan, China; 4 University of Chinese Academy of Sciences, Beijing 100049, China; 5 Key Laboratory of Plant Germplasm Enhancement and Specialty Agriculture, Wuhan Botanical Garden, Chinese Academy of Sciences, Wuhan 430074, China; 6 Administration of Shennongjia National Park, Shennongjia 442400, Hubei China; 7 Central Herbarium of Uzbekistan, Institute of Botany, Academy Sciences of Uzbekistan, Tashkent 100025, Uzbekistan

**Keywords:** anatomical, morphology, phylogeny, taxonomy

## Abstract

*Geum sunhangii* – first discovered in Shennongjia National Nature Reserve, Hubei Province, China – is described as a new species of Rosaceae. Compared to all known Chinese *Geum* species, the new species differs by possessing jointed styles, imbricate petals and a reniform radical leaf terminal leaflet. Most significantly, the jointed style is curved at an obtuse or a right angle. In addition, the inclusion of this species within the genus *Geum* was supported by phylogenetic analysis using the sequence data of a nuclear ribosomal internal transcribed spacer (nrITS) and a chloroplast *trnL–trnF* intergenic spacer. The new species was found to be closely related to *G. rivale* and *G. aleppicum*.

## Introduction

The genus *Geum* L. (1753: 500) (Rosaceae), also known as Avens, contains ca. 56 species distributed throughout the temperate, tropical and arctic regions of the world and is found mainly in the Northern Hemisphere (Gajewski 1959). The morphology of *Geum* is very complex. Most species are herbaceous perennials that form rosettes consisting of imparipinnate leaves and a thick caudex, but a few species are small shrubs. In addition, most species in this genus have fish-hook shaped fruits (Rafinesque 1833). Only three species *G. aleppicum* Jacq., *G. rivale* L. and G. japonicum var. chinense F.Bolle are present in China (Li et al. 2003). Bolle (1933) divided *Geum* into several genera, including *Novosieversia* F.Bolle and *Oncostylus* F.Bolle (Bolle, 1933), amongst others. However, based on cytological data, Gajewski (1957) concluded that *Geum* is a polyploid complex and did not support any previous circumscriptions of *Geum*; instead, Gajewski recognised most of the previously segregate genera as subgenera within the genus *Geum* (Gajewski 1957, 1968). Finally, his point of view was further supported by later morphological and molecular studies (Schulze-Menz 1964; Hutchinson 1967; Robertson 1974; Kalkman 1988; Smedmark and Eriksson 2002).

The Shennongjia National Nature Reserve is located in the northwest of China’s Hubei Province. It is a world diversity hotspot defined by its unique geographical location and complex topography (Hu et al. 2004). The Nature Reserve is also characterised by its high species diversity (including many rare and endangered animals and plants) and relict plant species (Fan et al. 2017). Correspondingly, it has recently attracted considerable attention from many researchers (Ma 2016; Xie et al. 2017). Recently, several new angiosperm taxa have been discovered there, including *Zhengyia shennongensis* T. Deng, D.G. Zhang & H. Sun (Deng et al. 2013), *Mazus sunhangii* D.G. Zhang & T. Deng (Deng et al. 2016) and *Impatiens baokangensis* Q.L. Gan & X.W. Li (Gan and Li 2016).

During a biodiversity survey of the Shennongjia National Nature Reserve, we discovered an undescribed species belonging to the Rosaceae. This species was distinguished by the presence of a rosette of basal leaves, petals and jointed styles. Our morphological and molecular studies revealed that the newly-collected material belongs to an unknown *Geum* species which is described here.

## Materials and methods

**Plant materials.** Specimens of the new species were collected from a site in Shennongjia National Nature Reserve, Hubei Province. Leaves of the new species were obtained for molecular studies. All herbaria used in this study were obtained from KUN (Herbarium, Kunming Institute of Botany, CAS).

**Anatomical observation.** Specimens of *G. sunhangii* were dissected and directly observed, before being placed under an anatomical lens for further observation under magnification. The cauline leaf, radical leaves, seeds, petal, style joint and stamen were inspected.

**DNA sequencing and molecular analyses.** Total DNA was extracted using a DP305 Plant Genomic DNA Kit (Tiangen, Beijing, China) and we selectively amplified the nuclear ribosomal internal transcribed spacer (nrITS) and chloroplast *trnL–trnF* intergenic spacer (*trnL–trnF*) regions by Polymerase Chain Reaction (PCR). Successfully amplified DNA fragments were then sequenced commercially. Molecular analysis was performed using two outgroups (i.e. *Fallugia paradoxa* Endl. and *Sanguisorba officinalis* L.; Potter et al. 2007), one piece of material from the putative new species and fourteen samples with similar morphology. Partial sequences were obtained from GenBank (https://www.ncbi.nlm.nih.gov/genbank); GenBank accession numbers for all species are listed in Table 2. Multiple sequence alignments were initially performed using CLUSTAL W ver. 1.4 (Thompson et al. 1994) and were manually adjusted using BioEdit ver. 7.0.5.3 (Hall 1999). We used SequenceMatrix ver. 1.7.8 to combine the sequences (Vaidya et al. 2011).

Phylogenetic trees were constructed using Maximum Parsimony (MP), Maximum Likelihood (ML) and Bayesian Inference (BI) algorithms. All characters were weighted equally, with gaps treated as missing data. MP analysis was conducted using PAUP ver. 4.0a (Swofford 2002). MP trees were obtained from a heuristic search of 1000 random addition replicates using tree bisection-reconnection (TBR). Finally, we obtained 1000 bootstrap values to evaluate the support for each branch.

ML analyses were conducted using the IQ–TREE web server (http://iqtree.cibiv.univie.ac.at/) (Trifinopoulos et al. 2016). We performed 1000 replicates with the substitution model set to ‘Auto’. Bayesian Inference was performed using Modeltest ver. 3.7 (Posada and Crandall 1998) to determine the best-fit models of nucleotide substitution. A comparison between the Akaike Information Criterion (AIC) values obtained with different tree models using the combined dataset revealed that TIMef was the most appropriate (Posada 2004). However, the best-fit TIMef model was substituted by the GTR + I model because TIMef was not available for further analyses. Bayesian Inference was performed using MrBayes ver. 3.1.2 (Ronquist 2003). The programme was run for 10 million generations and sampling was performed every 1000 generations. Each tree used two independent Markov Chain Monte Carlo (MCMC) (Yang 1997) analyses with four chains. When the average standard deviation of split frequencies was less than 0.01, a consensus tree was calculated after discarding the first 25% of trees as burn-in. A Bayesian tree was constructed from the remaining trees with Posterior Probability (PP) values for each clade.

## Results

### Taxonomic treatment

#### Geum sunhangii

Taxon classificationPlantaeRosalesRosaceae

D.G. Zhang, T. Deng, Z.Y. Lv & Z.M. Li sp. nov.

C0CE27B4-E648-5877-AF76-D216E0718005

urn:lsid:ipni.org:names:77211169-1

Figures 1 , 2

##### Type.

China. Hubei Province: Shennongjia National Nature Reserve, Nantianmen, alt. 2821 m, 11 July 2011, *zdg 7313* (holotype: KUM!)

**Figure 1.**
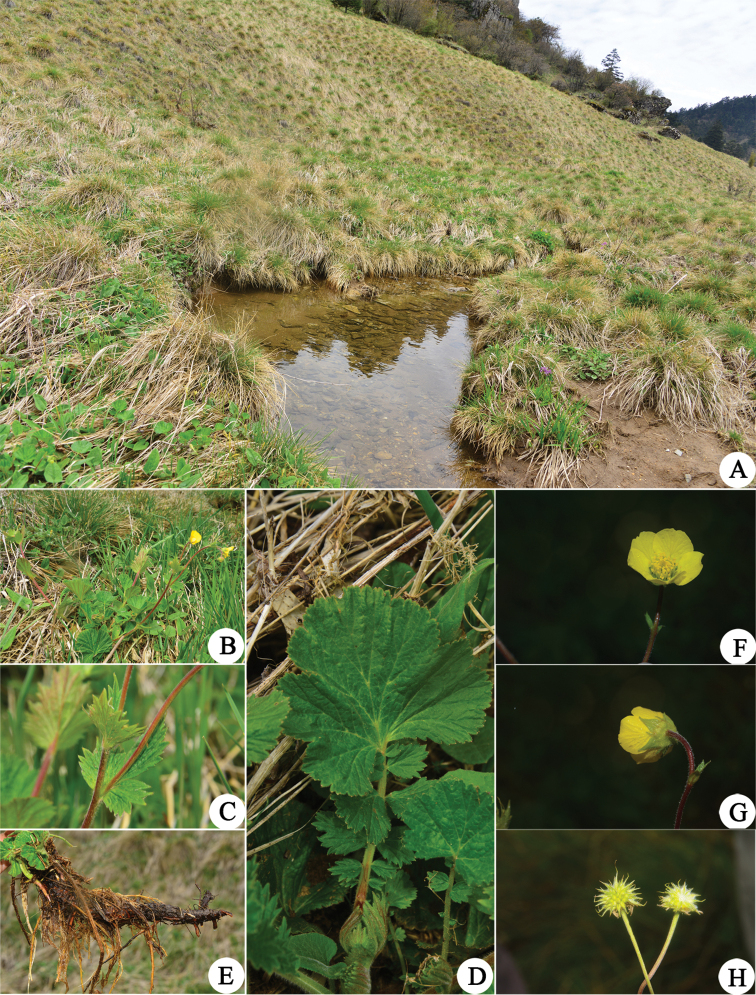
Images of *Geum sunhangii*. **A** Habitat **B** habit **C** cauline leaves **D** radical leaves **E** roots **F** corolla **G** sepals **H** infructescences.

##### Diagnosis.

*Geum sunhangii* and *G. aleppicum* are most similar in morphology. However, *G. sunhangii* differs significantly from *G. aleppicum* by the entire or 3-lobed cauline leaf (vs. blade pinnate, sometimes repeatedly lobed), reniform radical leaf terminal leaflet (vs. rhombic-ovate or compressed orbicular), nodding flowers (vs. erect), imbricate petals (vs. induplicate), curved at the obtuse or right angle at joint (vs. twisted), joint at ca. 2/3 of style apex (vs. ca. 1/4 way from apex). Detailed comparison information is in Table 1.

**Table 1.** Diagnostic morphological characters of *Geum sunhangii* compared to its three congeneric Chinese species.

	*G. sunhangii*	*G. aleppicum*	G. japonicum var. chinense	*G. rivale*
**Cauline leaf**	entire or 3-lobed	blade pinnate, sometimes repeatedly lobed	entire or 3-lobed	3-lobed or 3-parted
**Shape of radical leaf terminal leaflet**	reniform	rhombic-ovate or compressed orbicular	ovate or broadly obovate	rhombic-ovate
**Arrangement of petals**	imbricate	induplicate	induplicate	–
**Petal shape**	suborbicular	suborbicular	suborbicular	Semi-orbicular
**Petal colour**	yellow	yellow	yellow	yellow, purple-brown striate
**Sepal colour**	green	green	green	purplish
**Sepal growth mode**	spreading	spreading	spreading	erect
**Flower**	nodding	erect	erect	nodding
**Style joint**	obtuse or right angle curved	twisted	twisted	–
**Style joint location**	ca. 2/3 way from apex	ca. 1/4 way from apex	ca. 1/4 way from apex	–

**Table 2.** GenBank accession numbers for all species.

Species	Region	Accession	Region	Accession
***Geum sunhangii***	ITS	MT622526	*trnL–trnF*	MT614591
***Geum aleppicum***	ITS	KX645654	*trnL–trnF*	–
***Geum rivale***	ITS	AJ302352	*trnL–trnF*	AJ297338
***Geum andicola***	ITS	AJ302346	*trnL–trnF*	AJ297332
***Taihangia rupestris***	ITS	AJ302361	*trnL–trnF*	AJ297347
***Waldsteinia geoides***	ITS	AJ302362	*trnL–trnF*	AJ297348
***Coluria geoides***	ITS	AJ302343	*trnL–trnF*	AJ297329
***Geum bulgaricum***	ITS	AJ302347	*trnL–trnF*	AJ297333.
***Geum calthifolium***	ITS	MG235321	*trnL–trnF*	AB219633
***Geum heterocarpum***	ITS	AJ302349	*trnL–trnF*	AJ297335
***Geum vernum***	ITS	AJ302355	*trnL–trnF*	AJ297341
***Geum reptans***	ITS	AJ302351	*trnL–trnF*	AJ297337
***Geum montanum***	ITS	AJ302350	*trnL–trnF*	AJ297336
***Fallugia paradoxa***	ITS	U90805	*trnL–trnF*	AJ297331
***Sanguisorba officinalis***	ITS	KR052188	*trnL–trnF*	AJ416465

##### Description.

**Figure 2.**
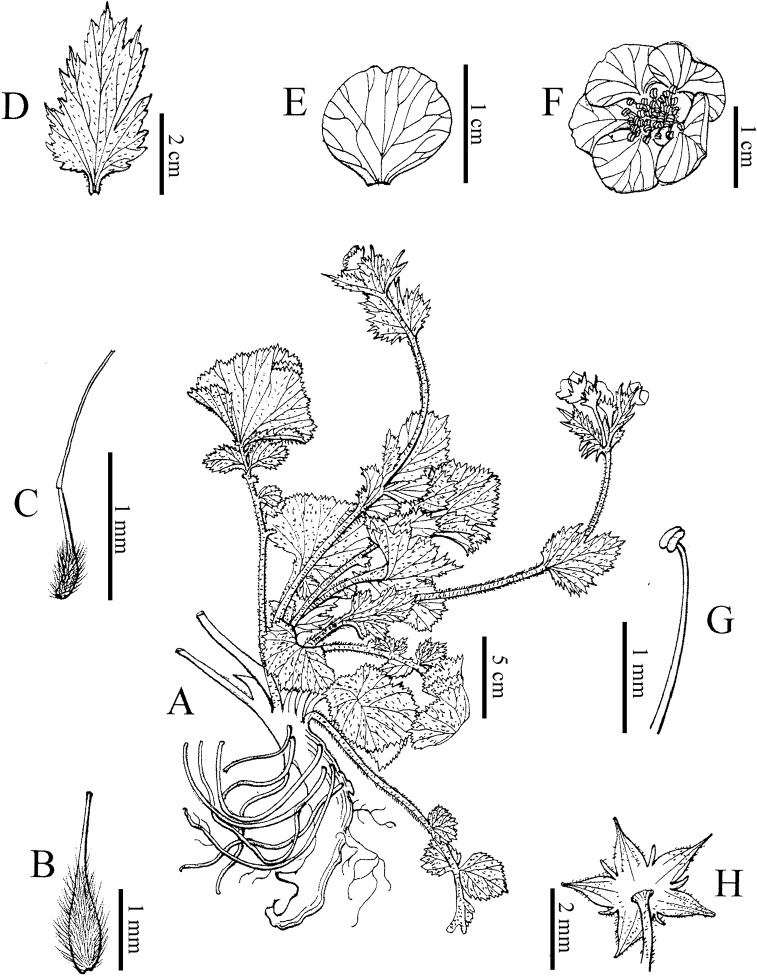
Holotype of *Geum sunhangii*. **A** Plant **B** achene **C** gynoecium **D** cauline leaf **E** petal **F** florets **G** filaments and anthers **H** calyx. Drawn by Ling Wang.

Roots fascicled, taproot terete, ca. 0.7 cm in diameter. Stems erect, 20–60 cm tall, pilose. Radical leaves lyrate-pinnate, 10–25 cm, with 2–4 pairs of leaflets, strigose; leaflets unequal, terminal leaflet largest, reniform, lobed, 2.5–6 × 3–10 cm, base cordate or truncate, margin irregularly coarsely serrate, apex rounded; cauline leaf ovate, herbaceous, 2–2.5 × 2.5–3.5 cm, leaf-like, leaf simple, entire or 3-lobed, margin irregularly coarsely serrate; veins bulge at leaf abaxially. Inflorescence terminal, corymb, bisexual, usually nodding, laxly 2–5 flowered. Flower actinomorphic, ca. 2.3 cm in diameter, pedicel densely pubescent and pilose; sepals triangular-ovate, green, apex acuminate; epicalyx elliptic or lanceolate, minute, ca. 1/3 as long as sepals, apex acuminate. Petals 5, yellow, imbricate, suborbicular, longer than sepals. Stamens numerous, yellow, ca. 0.22 cm; filament linear; anther yellow, ellipsoid. Style terminal, ca. 0.24 cm in length, curved at an obtuse or right angle at joint, joint at ca. 2/3 of style apex, style glabrous and apex separating from joint at fruit maturity. Infructescence ovoid or ellipsoid, ca. 80 seeds; fruiting receptacle hirtellous; achenes hirtellous, hair ca. 0.3 mm, erect (Figs 1, 2).

##### Phenology.

*G. sunhangii* flowers in May and sets fruit in August.

##### Distribution and habitat.

*Geum sunhangii* is currently found in Nantianmen, Shennongjia National Nature Reserve, Hubei Province (Fig. 6). It grows on hillside meadows near water.

##### Conservation status.

Based on the results of field investigations, *Geum sunhangii* was only discovered in Nantianmen, Shennongjia National Nature Reserve, where there is almost no human pressure on the distribution location of this species. About 60,000 individuals were present and the extent of occurrence is ca. 30,000 m^2^. It is possible that additional populations may be discovered during future excursions. We initially define the category of *G. sunhangii* as Near Threatened (NT) by the Guidelines for Using the IUCN Red List Categories and Criteria (IUCN 2017).

##### Vernacular name.

神农花, shén nóng huā in Chinese Pinyin.

##### Etymology.

The new species is named after the famous Chinese botanist Hang Sun, who made significant contributions to plant taxonomy, floral identification and biogeography in China.

### Phylogenetic analyses

We conducted a phylogenetic analysis of 15 species, based on nrITS and *trnL–trnF* sequence data. The aligned combined data matrix included 1649 characters and 397 variables. The three topologies inferred by the Bayesian Inference analyses, MP analyses and ML analyses were similar. The Bayesian tree, including MP bootstrap (BP), ML bootstrap (LP) and PP values, is presented in Figure 5. Bayesian analysis of the combined dataset showed that samples were divided into three clades. Clade III included most species of the genus *Geum*, while *Coluria geoides* (Pall.) Ledeb., *Waldsteinia geoides* Willd. and *Taihangia rupestris* T.T. Yu & C.L. Li were grouped in Clade II. Clade I was a sister clade to Clades II and III and consisted of only one species, *Geum andicola* Reiche. Finally, *G. sunhangii* was nested into a monophyletic group (PP = 1, LP = 100%, BP = 100%; Fig. 5) with *G. rivale*, *G. aleppicum* and *G. montanum* Gouan ex Steud. The new species was clustered with *G. rivale*, but with a weak support (PP = 0.82, LP = 73%, MP = 67%; Fig. 5).

## Discussion

**Figure 3.**
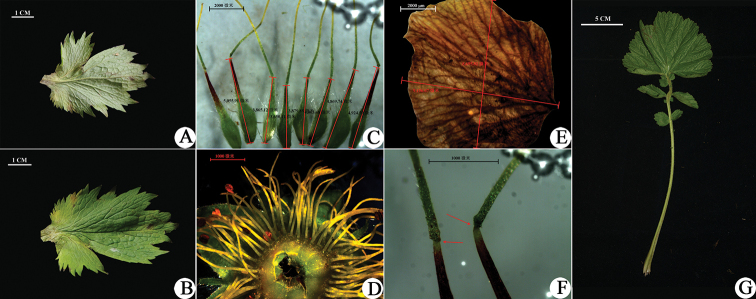
Anatomical characters of *Geum sunhangii*. **A, B** Cauline leaf **C** seeds **D** stamens **E** petal **F** style joints **G** radical leaf.

**Figure 4.**
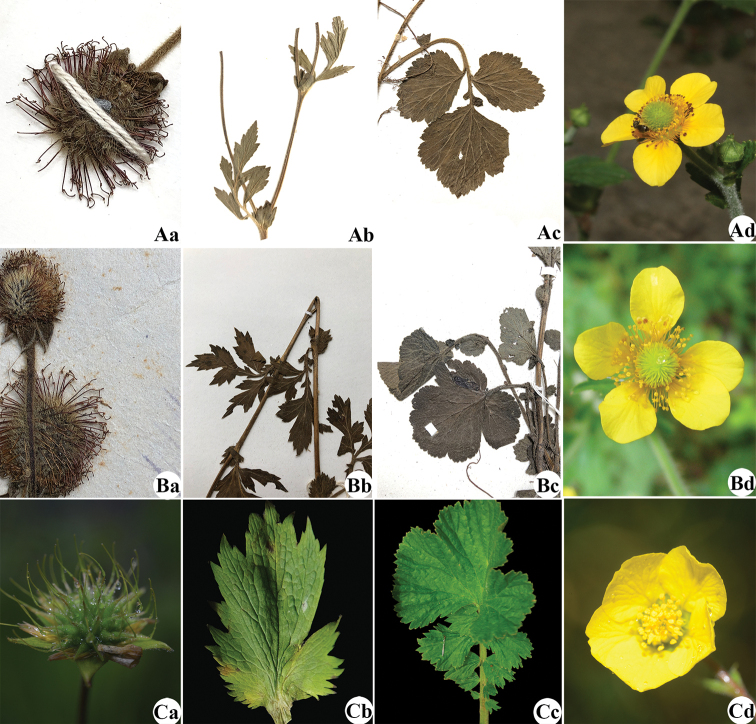
Morphological comparison of Chinese *Geum* species. **A***G. aleppicum***B**G. japonicum var. chinense**C***G. sunhangii* (infructescences (**a**), cauline leaves (**b**) radical leaves (**c**) and flowers (**d**)).

*Geum aleppicum* and G. japonicum var. chinense are similar in morphology; they both have similar yellow petals and green sepals, but these species differ in receptacle hair (Fig. 4Aa, Ba; Table 1). In contrast, *G. rivale* is easily recognisable due to its purplish sepals and purple-brown striped petals (Table 1). The nodding flower of the new species is similar to that of *G. rivale*. Therefore, the combination of green sepals, nodding flower, yellow and imbricate petals is distinctly different from those found in the other three species present in China. In addition, the radical leaf and style joint of the new species are distinct in this genus. The terminal largest leaflet of the radical leaf is reniform (Fig. 3G) and different from the other species, which possess rhombic-ovate or compressed orbicular leaflets (Table 1). Curved styles are also important and recognisable features of the new species. Obtuse or right-angle curves of the style joint can be used to distinguish between the new species and its close relatives (Figs 3F, 4a). All distinguishing features are shown in Fig. 4 and Table 1.

**Figure 5.**
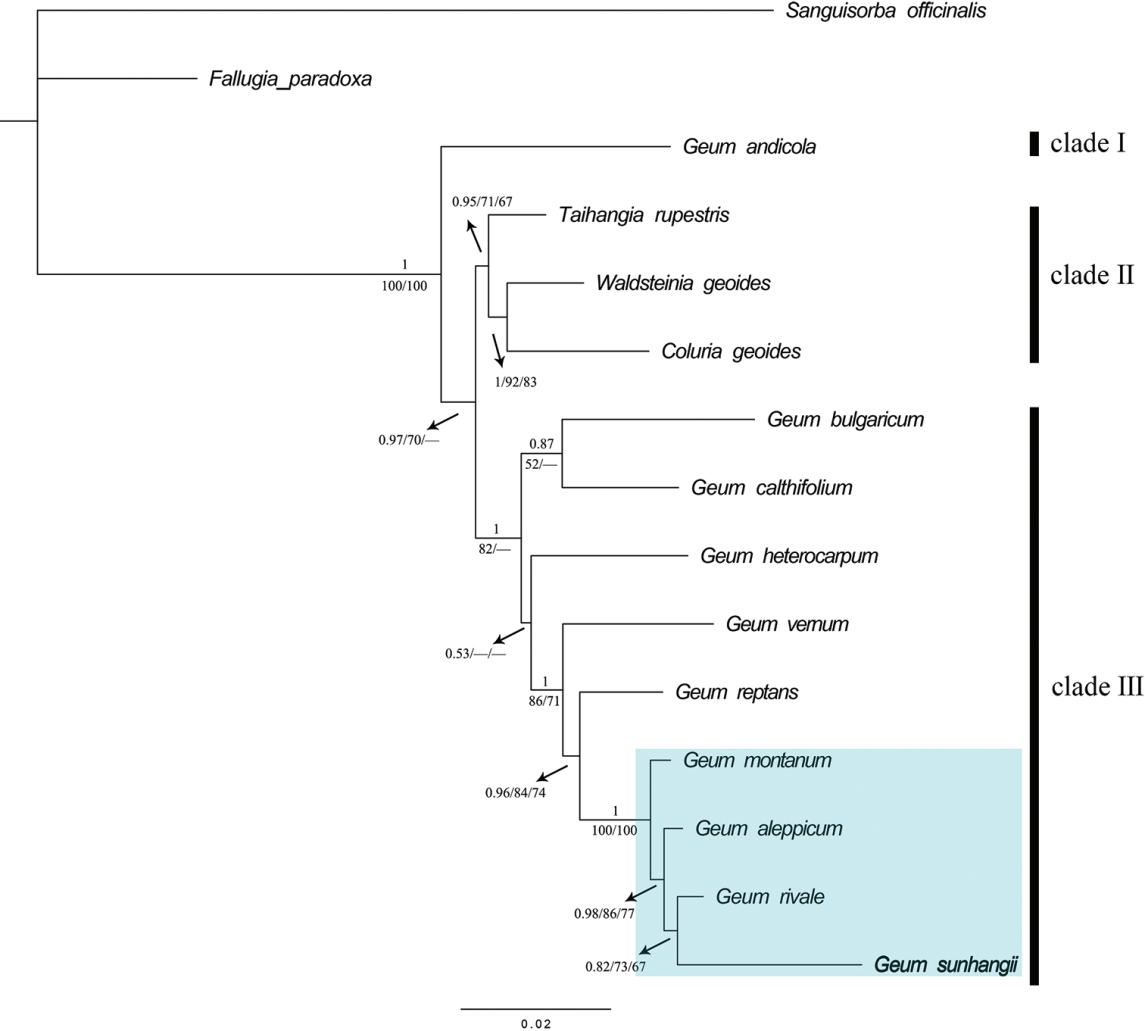
Bayesian consensus tree of the combined ITS and *trnL–trnF* sequence dataset. Numbers above branches indicate Bayesian posterior probability [PP], numbers below branches represent maximum likelihood bootstrap [LP] and maximum parsimony bootstrap [BP] values. The dash (–) indicate BP and LP < 50%. The new species is shown in bold. The monophyletic group with high support is framed by the blue rectangle.

We obtained samples of fifteen species for phylogenetic analysis. The topologies of the Bayesian, ML and MP trees were identical and were consistent with previous studies (Smedmark and Eriksson 2002). According to our results, *Geum* is probably a polyphyletic group and the boundary between *Geum* and species in Clade II is not clear (Fig. 5). At the same time, species in the genera *Taihangia* T.T. Yu & C.L. Li, *Coluria* R.Br. and *Waldsteinia* Willd. are likely congeneric to the *Geum* species. Therefore, further studies of the taxonomic and phylogenic relationships of *Geum*, *Taihangia*, *Coluria* and *Waldsteinia* species are needed. At present, the new species was confirmed as a member of *Geum*, since *G. sunhangii* was nested within a group of *Geum* species (PP = 1, LP = 100%, MP = 100%; Fig. 5) that form a monophyletic group (Fig. 5). In addition, the new species can be easily identified by its morphological features.

**Figure 6.**
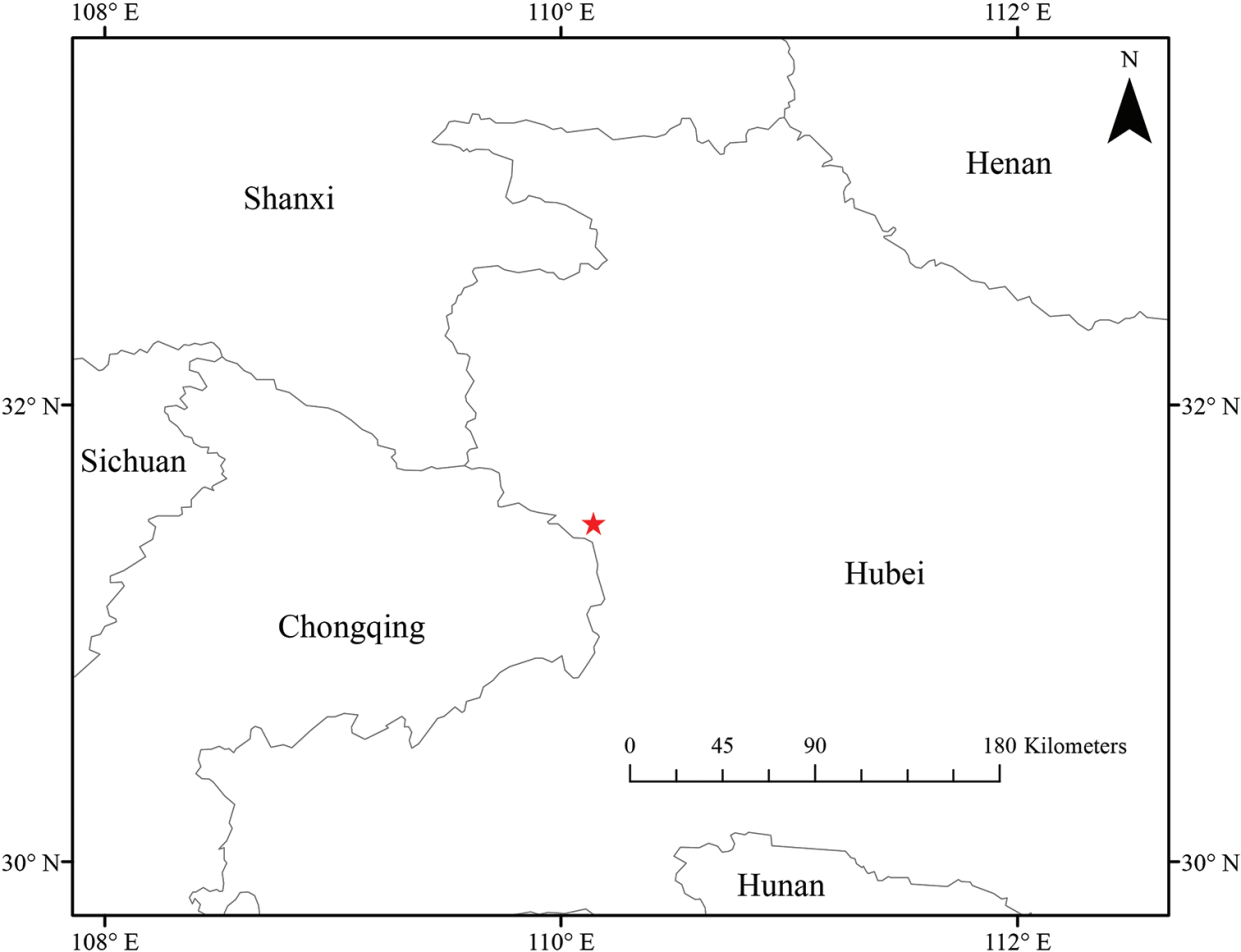
Distribution of *Geum sunhangii* in Hubei Province, China. Star indicates the type locality of *G. sunhangii*.

## Supplementary Material

XML Treatment for Geum sunhangii
